# Supplementary material for: Positive Correlation of the Gene Rearrangements and Evolutionary Rates in the Mitochondrial Genomes of Thrips (Insecta: Thysanoptera)
Source: Insects. 2022 Jun 27;13(7):585. doi: 10.3390/insects13070585 (PMC9321049; doi:10.3390/insects13070585)
Supplement: Supplementary file 1 [file insects-13-00585-s001.zip › insects-1765699-supplementary.pdf]

Table S1. Features of the mitogenome of *Aeolothrips xinjiangensis*.

Gene	Direction	Location	Size (bp)	Anticodon	Codon Start	Codon Stop	Intergenic nucleotides
COI	F	1-1560	1560		ATA	TAG	0
trnS1	F	1560-1624	65	TCT			-1
srRNA	F	1625-2409	785				0
ATP8	F	2410-2574	165		ATC	TAG	0
ATP6	F	2571-3252	684		ATG	T	-4
trnK	F	3253-3318	66	TTT			-2
ND1	F	3382-4301	920		ATT	TA	63
trnL2	F	4302-4367	66	TAA			0
trnW	F	4366-4431	66	TCA			-2
trnR	F	4441-4512	72	TCG			9
CR1		4513-6106	1594				0
COII	F	6107-6778	672		ATA	TAA	0
trnL1	F	6808-6874	67	TAG			29
COIII	F	6906-7694	789		TTG	TAA	31
ND3	F	7701-8051	351		ATG	TAA	6
trnN	F	8064-8131	68	GTT			12
trnE	F	8130-8199	70	TTC			-2
trnQ	F	8222-8289	68	TTG			22
trnI	F	8291-8358	68	GAT			1
CYTB	F	8361-9491	1131		ATA	TAG	2
trnG	F	9491-9563	73	TCC			-1
CR2		9564-9919	356				0
ND2	R	9920-10963	1044		ATA	TAA	0
trnC	R	10964-11026	63	GCA			0
trnY	R	11029-11095	67	GTA			2
ND5	R	11094-12800	1707		ATT	TAA	-2
trnH	R	12801-12865	65	GTG			0
ND4	R	12869-14191	1323		ATG	TAA	3
ND4L	R	14340-14624	285		ATA	TAA	148
trnT	F	14643-14703	61	TGT			18
trnP	R	14749-14815	67	TGG			45
ND6	F	14818-15321	504		ATA	TAA	2
trnM	F	15322-15386	65	CAT			0
trnA	F	15387-15452	66	TGC			0
trnF	F	15453-15518	66	GAA			0
trnV	F	15521-15587	67	TAC			2
lrRNA	F	15588-16806	1219				0
trnS2	F	16807-16876	70	TGA			0
trnD	F	16882-16947	66	GTC			5

Note: Intergenic sequences refer to intergenic regions between each gene and the gene after it.

Table S2. Codon usage of 13 PCGs in the mitogenome of *Aeolothrips xinjiangensis*.

Codon	Count	Codon	Count	Codon	Count	Codon	Count
**UUU(F)**	**376**	**UCU(S)**	**139**	**UAU(Y)**	**123**	**UGU(C)**	**60**
UUC(F)	49	UCC(S)	29	UAC(Y)	36	UGC(C)	5
**UUA(L)**	**297**	UCA(S)	88	UAA (*)	8	**UGA(W)**	**88**
UUG(L)	42	UCG(S)	13	UAG (*)	3	UGG(W)	9
CUU(L)	91	**CCU(P)**	**56**	**CAU(H)**	**51**	**CGU(R)**	**11**
CUC(L)	10	CCC(P)	16	CAC(H)	11	CGC(R)	0
**CUA(L)**	**50**	CCA(P)	48	**CAA(Q)**	**47**	CGA(R)	34
CUG(L)	10	CCG(P)	7	CAG(Q)	3	CGG(R)	4
**AUU(I)**	**309**	**ACU(T)**	**62**	**AAU(N)**	**138**	AGU(S)	27
AUC(I)	29	ACC(T)	19	AAC(N)	34	AGC(S)	1
**AUA(M)**	**226**	ACA(T)	84	**AAA(K)**	**117**	**AGA(S)**	**65**
AUG(M)	38	ACG(T)	2	AAG(K)	20	AGG(S)	12
**GUU(V)**	**103**	**GCU(A)**	**66**	**GAU(D)**	**57**	GGU(G)	49
GUC(V)	9	GCC(A)	13	GAC(D)	10	GGC(G)	2
GUA(V)	85	GCA(A)	40	**GAA(E)**	**87**	**GGA(G)**	**140**
GUG(V)	13	GCG(A)	4	GAG(E)	17	GGG(G)	18

NOTE. AA indicates amino acid. tRNAs are represented by the IUPAC-IUB single letter amino acid codes. N is the number of times of a codon is used. The asterisk (*) indicates a termination codon. Values in bold type stand for the most commonly used codon for amino acid. Underlined stand for the cognate codon of tRNA for each amino acid.

TableS3. Comparison of the degree of gene rearrangement (breakpoints), non-synonymous substitution rates (Ka) and intergenic nucleotides between *ND4* and *ND4L* in the mitogenomes of fourteen thrips.

Species	Ka	Breakpoints	Branch length (Mya)	Intergenic nucleotides (*ND4-ND4L*)
Haplothrips aculeatus	0.501	31	6.63	-7
Gynaikothrips uzeli	0.506	33	7.06	-7
Franklinothrips vespiformis	0.326	29	3.6	-7
Aeolothrips xinjiangensis	0.453	28	3.91	148
Holarthrothrips indicus	0.351	25	3.04	-1
Rhiphiphorothrips cruentatus	0.417	25	4.4	-7
Dendrothrips minowai	0.416	28	4.23	10
Anaphothrips obscurus	0.421	29	4.45	-7
Thrips palmi	0.414	31	4.44	-7
Thrips imaginis	0.417	30	4.67	-21
Franklinithrips occidentalis	0.422	30	4.73	18
Franklinithrips intonsa	0.435	30	4.79	-7
Neohydatothrips samayunkur	0.446	33	5	-7
Scritothrips dorsalis	0.427	30	4.46	5

NOTE. The represents (+) values as intergenic nucleotides and (-) values as overlapping regions of intergenic nucleotides.

Table S4. Normality test for the Ka values, breakpoint values, and branch lengths in the phylogenetic tree.

Data	Statistical magnitude	*df*	*p*
Ka	0.899	14	0.109
Breakpoints	0.920	14	0.221
Branch length	0.868	14	0.039

NOTE. *df* is the sample size. *p*, >0.05 (conforms to normal distribution) and > 0.05 (not conforms to normal distribution).

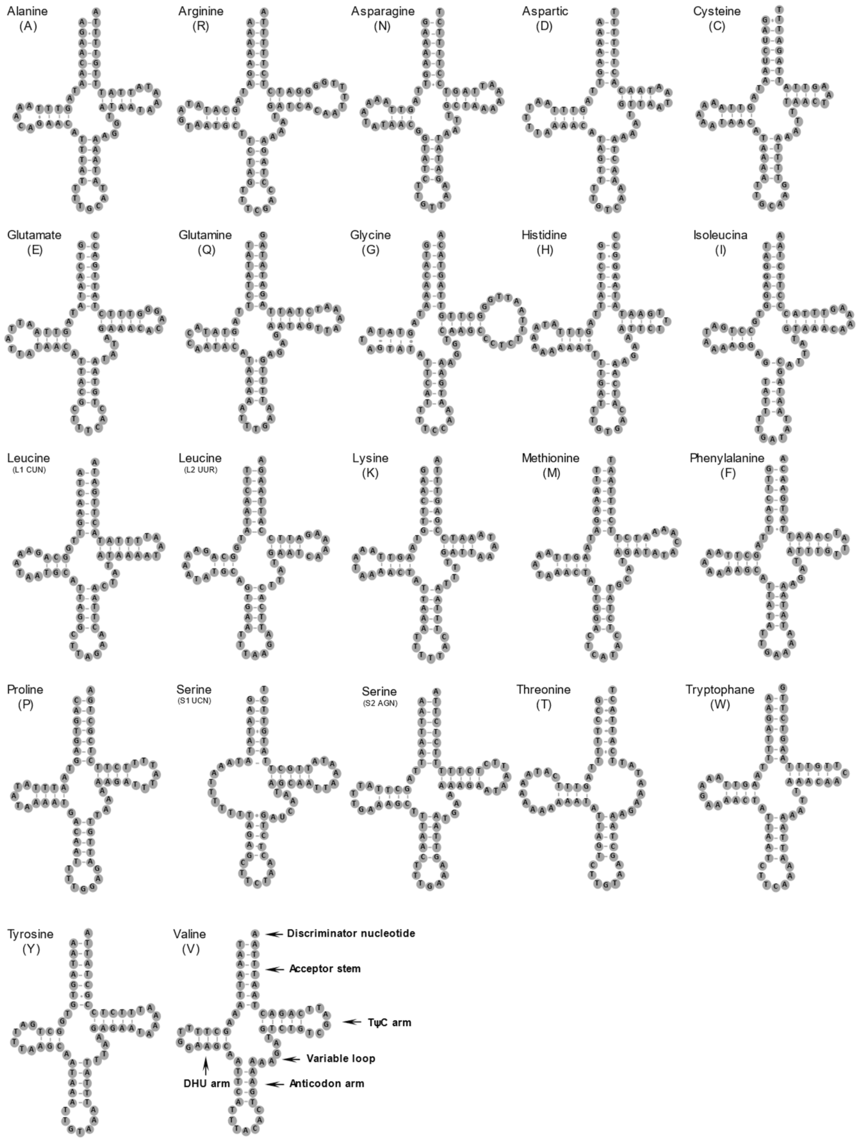

Figure S1. The secondary structure of 22 tRNAs. The tRNAs are labeled with the abbreviations of their corresponding amino acids. Inferred Watson-Crick bonds are illustrated by lines, while the mismatches are illustrated by dots.
